# Miniaturized systems for evaluating enzyme activity in polymeric membrane bioreactors

**DOI:** 10.1002/elsc.201900059

**Published:** 2019-10-09

**Authors:** Mohammad S. Islam, Cindy K. Harnett

**Affiliations:** ^1^ Department of Electrical and Computer Engineering University of Louisville Louisville KY USA

**Keywords:** enzyme immobilization, laccase, lignin degradation, polymeric membrane bioreactor

## Abstract

Enzyme‐coated polymeric membranes are versatile catalysts for biofuel production and other chemical production from feedstock, like plant biomass. Such bioreactors are more energy efficient than high temperature methods because enzymes catalyze chemical reactions near room temperature. A major challenge in processing plant biomass is the presence of lignin, a complex aromatic polymer that resists chemical breakdown. Therefore, membranes coated with enzymes such as laccase that can degrade lignin are sought for energy extraction systems. We present an experimental study on optimizing an enzyme‐based membrane bioreactor and investigate the tradeoff between high flow rate and short dwell time in the active region. In this work, zero flow rate voltammetry experiments confirm the electrochemical activity of *Trametes versicolor* laccase on conductive polymer electrodes, and a flow‐through spectroscopy device with laccase‐coated porous nylon membranes is used with a colorimetric laccase activity indicator to measure the catalysis rate and percent conversion as a function of reactant flow rate. Membrane porosity before and after laccase coating is verified with electron microscopy.

**Abbreviations**
ABTS: 2,2′‐azino‐bis(3‐ethylbenzthiazoline‐6‐sulfonic acid)
SPE: screen‐printed electrodes

## 1 INTRODUCTION

The major challenge associated with processing plant biomass is the presence of lignin, a hydrophobic and heterogeneous biopolymer that resists chemical and biological degradation. It is the second most abundant naturally synthesized compound after cellulose comprising 15–40% of dry weight in most plants 1. It is a complex aromatic polymer rich in phenolic compounds; enzyme‐based reactors that can oxidize these phenols and depolymerize lignin could provide a new route to sustainable biofuels and aromatic fine chemicals beyond conventional refineries 2. Research groups are looking at laccase enzymes for breaking down organic materials 3; these enzymes can adhere to charged membrane surfaces and can also be engineered with binding sites such as his‐tag sequences to attach to metal‐ion coated synthetic membranes. Membrane‐immobilized enzymes that can break down lignin from woody and non‐woody plants are sought after for compact and room‐temperature energy extraction systems. In this work, a flow‐through device was developed with a goal of evaluating genetically engineered enzymes’ activity in a microreactor environment using a membrane‐based lab‐on‐chip sensor system. The small‐scale screening system is designed to test sub‐100 microgram quantities of enzymes in a membrane reactor format so the most active variants can be identified for scale‐up.

**Figure 1:**
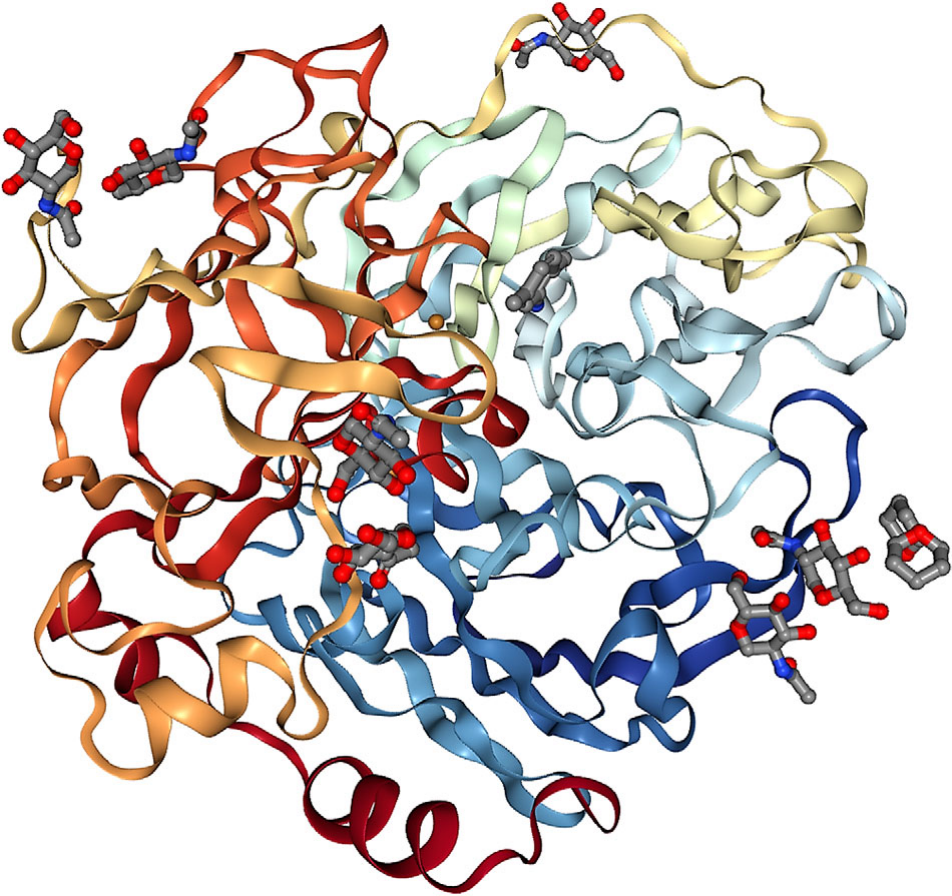
Active laccase enzyme from *Trametes versicolor* complexed with 2,5‐xylidine 23

Laccase, an environmentally friendly and functionally diverse enzyme, is well known for its lignolytic activity. It is produced by a variety of fungi, bacteria, and plants 4, 5, and serves as an efficient catalyst for bioremediation, due to its ability to catalyze the oxidation of a variety of substrates, such as phenolic compounds, metal ions, and aromatic amines 6. On account of its relatively low redox potential (450–800 mV), laccase cannot directly catalyze the oxidation of most nonphenolic substrates (e.g. 80% of lignin). But its substrate scope can be widened to nonphenolic compounds once combined with low molecular weight mediators, which simultaneously act as substrates for the enzyme 7, 8, 9. As laccase's range of substrates have broadened in the last couple of decades, so has its influence in bioremediation applications, wood pulping, paper and textile industry, municipal sewage, electrochemical analysis, and organic synthesis applications 10, 11, 12, 13, 14, 15, 16, 17. The range of compounds identified over the decades as mediators for the laccase‐mediator system has increased dramatically. Currently, 2,2′‐azino‐bis(3‐ethylbenzthiazoline‐6‐sulfonic acid) (ABTS) and m‐cresol are currently regarded as the best mediators for laccase 18, 19, 20. For this work, *T. versicolor* laccase shown in Figure 1 was employed due to its promising activity in bioremediation presented in ref. 21, 22. The developed enzyme screening system seeks to work with sub‐milligram laccase quantities produced in early‐stage genetic engineering research. Voltammetric analysis performed in the presence of m‐cresol isomer determined the enzyme activity without flow. A flow‐through spectroscopy device with enzyme‐coated membranes and ABTS, which is a well‐characterized colorimetric indicator for spectroscopy, was employed to measure the percentage of conversion as a function of flow rate and membrane microstructure.

## 2 MATERIALS AND METHODS

### 2.1 Materials

All chemicals used during the laboratory‐scale membrane fabrication and other studies were of reagent grade and used without further purification. Potassium chloride, potassium phosphate monobasic, potassium phosphate dibasic, sodium acetate, acetic acid, m‐cresol, ABTS, and the enzyme laccase from *T. versicolor* (powder, light brown, ≥0.5 U/mg) were purchased from Sigma‐Aldrich.

**PRACTICAL APPLICATION**

Enzyme‐catalyzed membrane reactors offer environmentally friendly, compact, and energy efficient replacements for chemical production and energy generation. However, the best combination of enzyme variant, membrane structure, membrane coating, reactant concentration, and flow rate must be identified through modeling and experiment. One of the key variables is the residence time of reactants near the membrane‐bound enzymes. A long dwell time is ideal for high conversion percentage, but not for fast production rates. Because the colorimetric and voltammetric assays in this work only require millimeter volumes of outflow, it is practical to explore a wide range of residence times. Another important variable is the enzyme itself. An enzyme such as laccase has hundreds of variants that evolved for optimal activity in different organisms with different environmental conditions. This microscale system is a platform for evaluating sub‐milligram quantities of genetically engineered enzymes in a membrane reactor environment before investing in production and scale‐up.

Several polymer membranes were evaluated for absorbance measurement experiments as presented in Table 1. Supercharged nylon filters (Whatman Nytran SPC, 0.45 µm pore size) were obtained from Tisch Scientific; this treated nylon has a high positive charge per area for picking up protein in blotting assays. Thicker than other membranes in Table 1, the Nytran material can also lie flat without curling, for easier coating and assembly into flow‐through systems.

**Table 1:** Survey of commercially available polymer membranes for flow‐through enzyme reactor system

Membrane	Pore size (µm)	Manufacturer	Nominal thickness (µm)	Binding capacity (µg/cm^2^)
Polyethersulfone	1.2	Sterlitech	110–150	20
Polycarbonate	0.2	Sterlitech	10	5
Polyester	0.2	Sterlitech	10	< 5
Polyvinylidene difluoride	0.2	Sterlitech	125	4
Nylon	0.2	Sterlitech	65–130	120
Nytran SPC Nylon	0.45	Tisch Scientific	140–170	600

Voltammetric measurements were conducted using three‐electrode configured screen‐printed electrodes (SPE) from Dropsens Inc. (product no. P10). These disposable electrodes are constructed on a flexible plastic substrate consisting a PEDOT working electrode, a carbon counter electrode, and a silver reference electrode.

### 2.2 Electrochemical detection of immobilized enzyme activity

**Figure 2:**
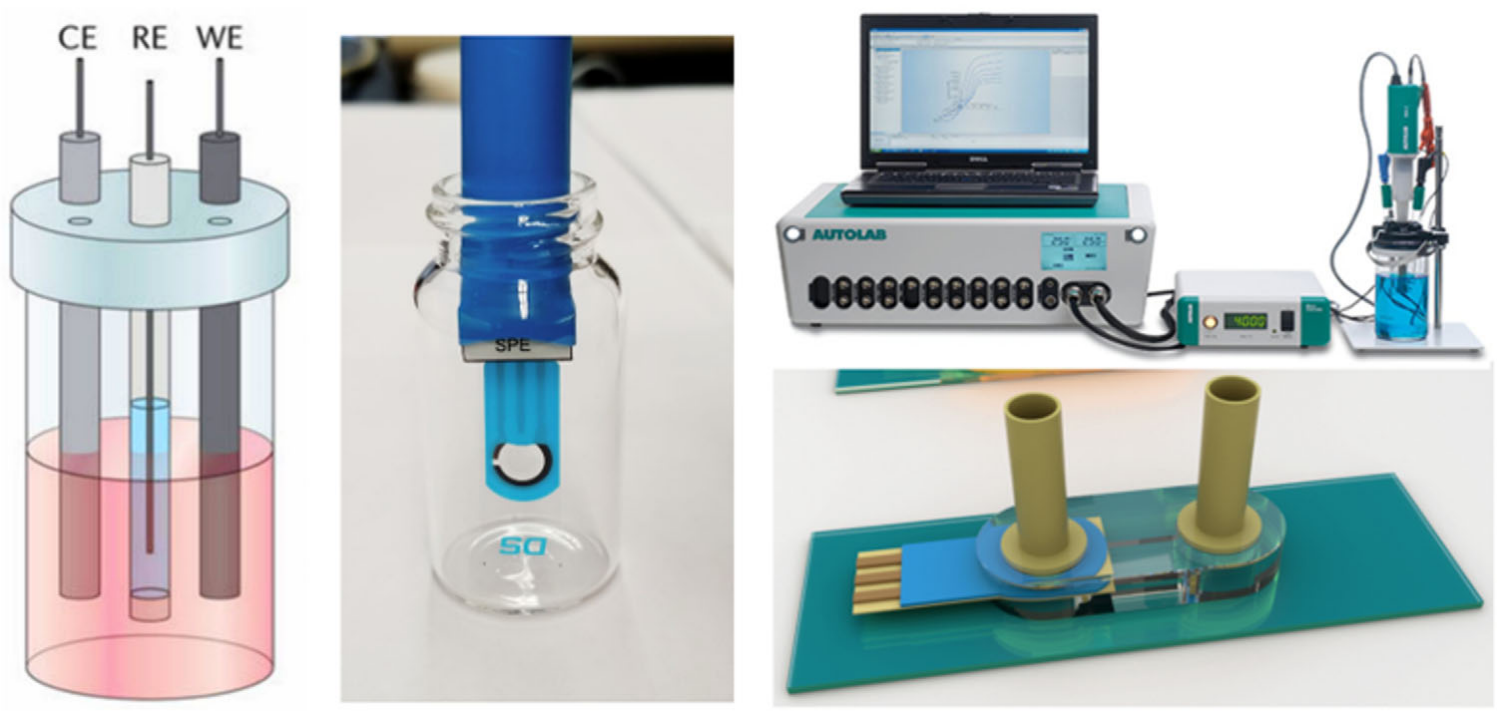
A typical experimental setup showing the reference, counter, and working electrodes (left); setup employed for in‐lab experiments with SPEs (middle); Metrohm Autolab N series potentiostat (top‐right); design of a flow‐through environment using a membrane‐based lab‐on‐chip sensor system (bottom‐right)

Cyclic voltammetry provides direct information about the redox properties of laccase by monitoring currents into and out of electrode‐bound enzyme coatings. Analysis of the resulting electrochemically active products is another source of data about enzyme activity. Such studies could potentially evaluate the quality and lifespan of laccase‐based enzyme biosensors and bioreactors. To demonstrate the feasibility of the proposed approach in a small‐volume format, cyclic voltammetry analysis of m‐cresol was performed at bare and laccase‐modified SPE as illustrated in Figure 2.

Cresols are acquired from antioxidants such as phenolic acids. Although m‐cresol is a small molecule, its structure resembles lignin's phenolic sub‐units, making it a convenient substrate for modeling lignin breakdown. Cresol isomers along with other polyphenolic compounds show electrochemical activity in the presence of laccase and also possess significant antioxidant properties beneficial for human health 20.

Voltammetric measurements were carried out using Metrohm Autolab N series potentiostat/galvanostat models 128N and 302N, which were controlled by the latest version of NOVA software. The SPEs were soaked in laccase solution (*T. versicolor* laccase, Sigma‐Aldrich, 3 mg/mL in pH 7 0.5 M potassium phosphate buffer with 100 mM KCl) overnight and dried for 4–5 h afterwards to develop the coating. The analysis was carried out with m‐cresol concentration of 5 × 10^–4^ mol/L in a 0.01 mol/L non‐deaerated acetate buffer (pH 5.0) as the supporting electrolyte. The recipe for acetate buffer (0.01 M 500 mL) consists of sodium acetate solution (0.01 M 352.5 mL), acetic acid solution (0.01 M 147.5 mL), and 27 mg of m‐cresol. The potential range for the cyclic voltammetry was set from −0.4 to +1.0 V and scan rates of 5–20 mV/s were used.

### 2.3 Immobilizing enzymes on membranes for colorimetric detection

Nytran SPC membrane sheets were laser‐cut into 1 cm diameter discs for laccase coating. The laccase stock solution recipe consisted of the following: potassium chloride (200 mM 50 mL), potassium phosphate monobasic (1 M 19.25 mL), potassium phosphate dibasic (1 M 30.75 mL), and 300 mg laccase (*T. versicolor*) powder; for 100 mL of 0.5 mM pH 7 solution. The laccase buffer solution was divided into 1 mL centrifuge tubes and flash‐frozen within a day to keep the protein fresh; tubes were thawed one at a time for experiments. Nytran membranes were then functionalized with laccase enzyme via a drop casting method; each membrane disc was exposed to 20 µL drops of the laccase solution from a pipette. The membranes were left to dry for 5–6 h.

**Figure 3:**
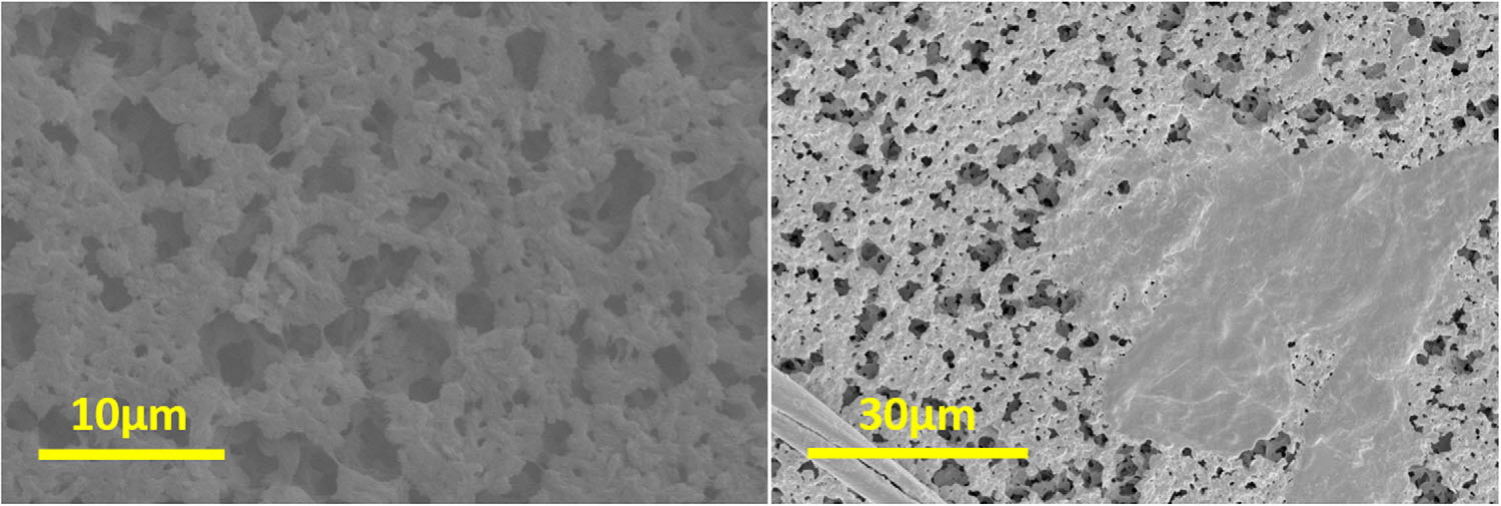
SEM images of the Nytran SPC membrane — plain membrane (left) and with immobilized laccase (right)

**Figure 4:**
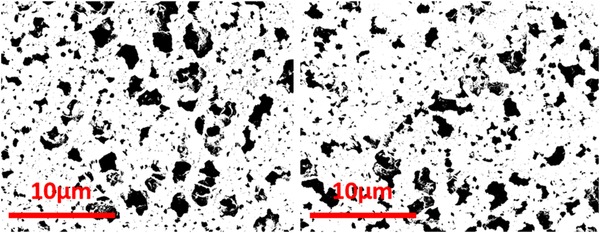
SEM images of the Nytran SPC membrane under similar magnification — plain membrane (left) and after enzyme immobilization (right); smaller pores are filled on the enzyme‐coated membrane

The enzyme immobilization was further confirmed by colorimetric analysis of ABTS solution (detailed further in Section 2.5) exposed to laccase‐coated membrane discs by dipping and scanning electron microscopic analyses. Comparison of the as‐received Nytran SPC membrane and the same membrane after coating with laccase is shown in Figure 3. Scanning electron micrographs revealed surface films (Figure 3, right) that were not present on as‐received membranes, which are later washed away when the membrane placed inside the microreactor is rinsed with DIUF (de‐ionized ultra‐filtered) water. The SEM images of the plain and enzyme coated membranes were evaluated further to determine the porosity of the membranes. In Figure 4, image analysis of the plain membrane (left) yields a 19% porosity whereas the enzyme‐exposed membrane has a lower porosity of 16%. The plain membrane has a greater fraction of open area compared to that of the enzyme exposed membrane, suggesting the enzyme coating, which includes crystallized buffer salt, is covering and filling the smaller pores and reducing the open area.

After the enzyme immobilization step, the membrane was placed between laser cut acrylic pieces and glued together to form the flow‐through reactor (detailed further in Section 2.4). The membranes were then rinsed thoroughly by flowing DIUF water through the reactor with a syringe pump to wash off loosely‐bound enzymes. This process was carried out with several (2–4) 20 mL aliquots of DIUF water. Removing loosely‐bound enzyme promotes a steady‐state situation in the membrane reactor by keeping the enzyme surface density constant during testing, and by preventing enzymes from falling into suspension and catalyzing reactions downstream. Aliquots from the permeate were tested for the presence of free laccase enzyme by exposing it to ABTS solution. It was observed that the amount of leached enzyme was negligible after the third wash as the ABTS did not change color. These enzyme‐coated membranes were used with the colorimetric detection method described below in Section 2.5.

### 2.4 Design and fabrication of a microstructured membrane bioreactor

**Figure 5:**
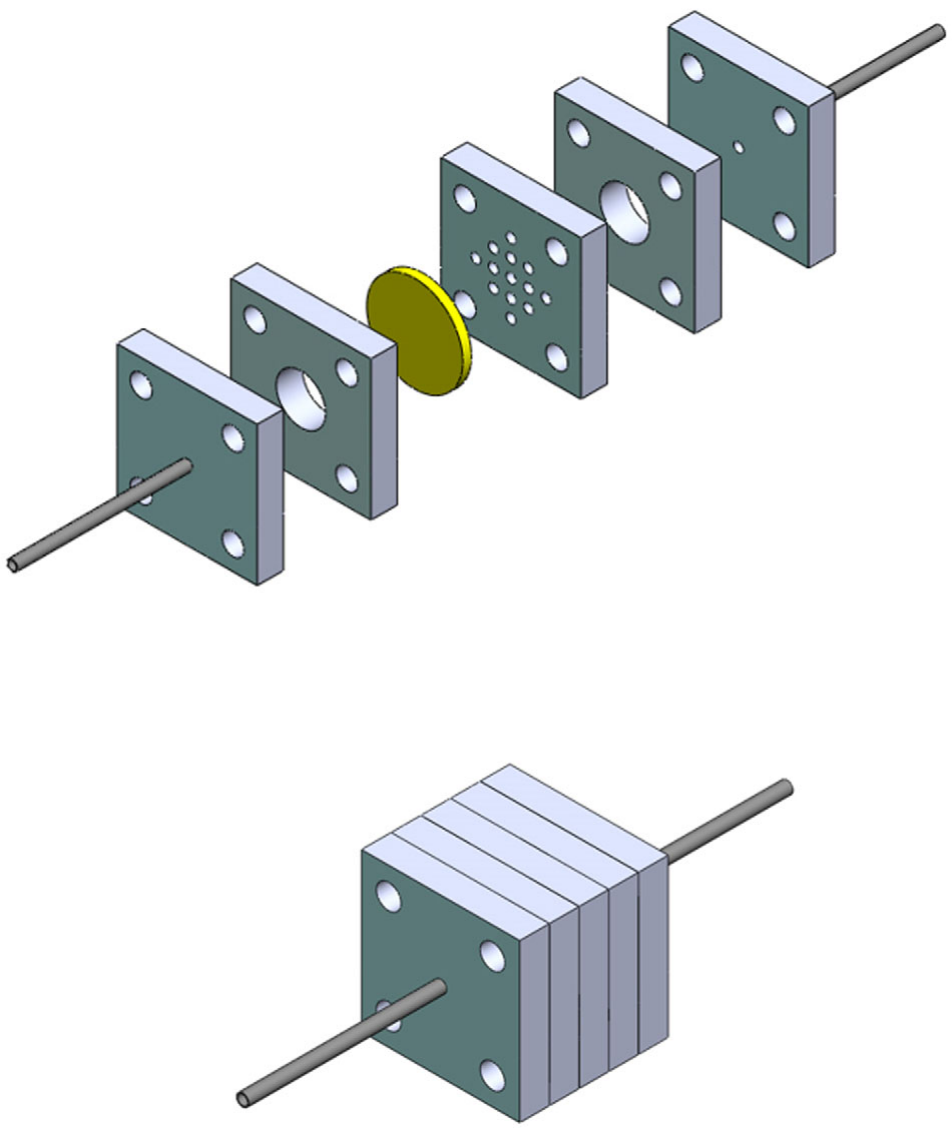
Schematic of the laser cut acrylic flow‐through device. Membrane (yellow disc) is sandwiched between the pieces and glued

**Figure 6:**
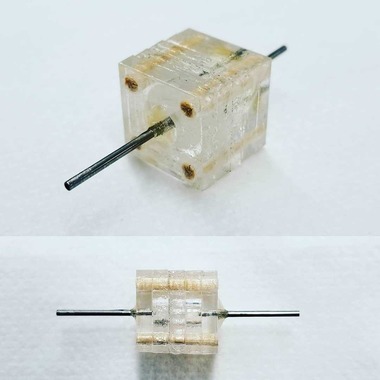
Assembled flow‐through laccase‐coated membrane device

To demonstrate colorimetric detection of the immobilized enzyme in a flow‐through format, a plastic housing was designed to drive convective flow through membranes. The flow‐through device consisted of a functionalized membrane sandwiched between several acrylic laser‐cut pieces (3 mm thick) which served as scaffolding to hold the membrane in place and as reservoirs for the flow medium. Two stainless steel hypodermic tubes (Ziggy's Tubes and Wires part no. 18R316‐0.787) connected silicone tubing (McMaster Carr 51845K52) to a syringe pump at the inlet, and a spectrometer cuvette at the outlet. The individual pieces were glued together with epoxy potting compound (3M Scotch‐Weld DP270). A breakdown of the flow‐through device can be seen in Figures 5 and 6.

### 2.5 Optical absorbance measurement

**Figure 7:**
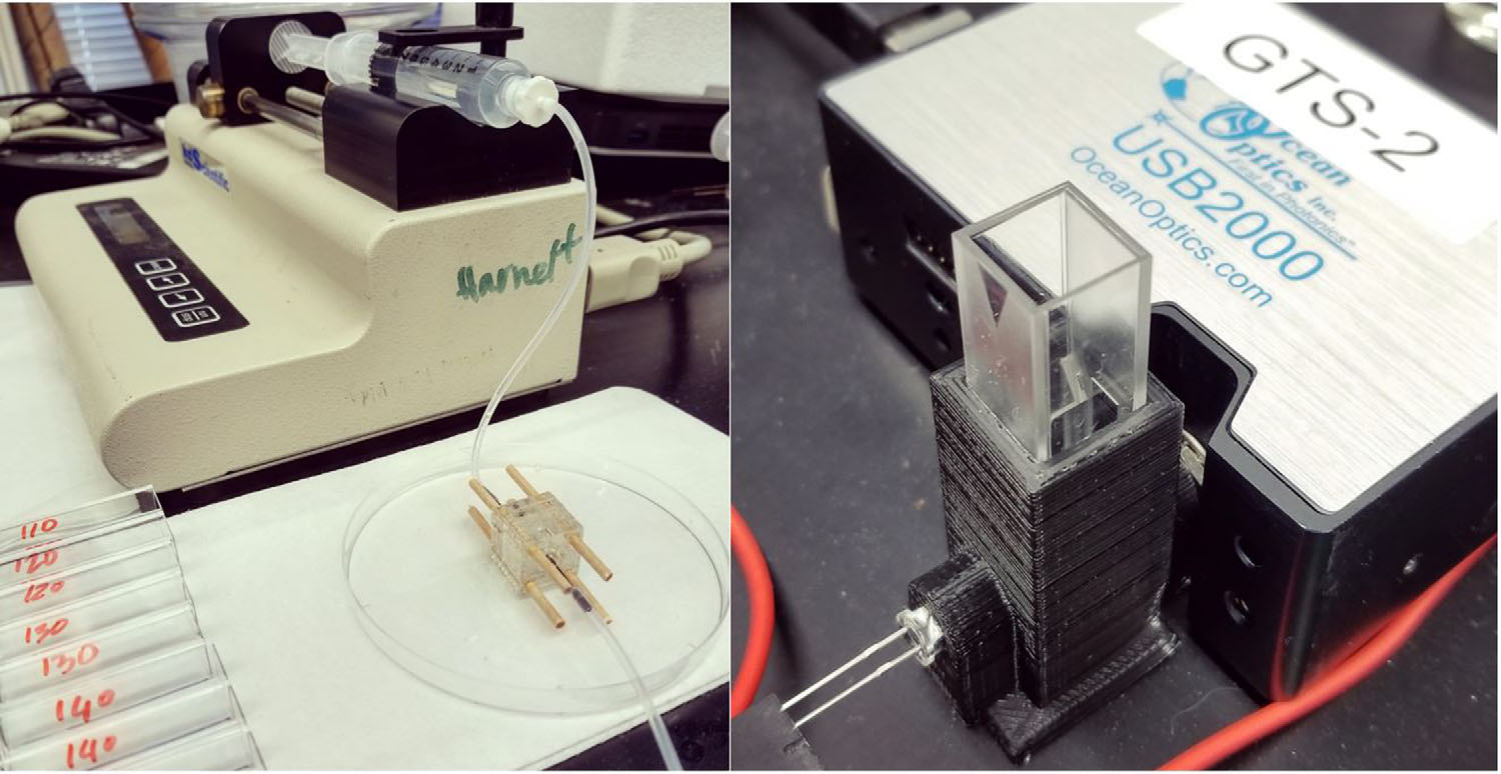
Flow‐through reactor connected to a syringe pump (left); cuvette containing reacted ABTS solution analyzed by a spectrophotometer (right)

ABTS is a chemical compound used to observe enzyme reaction kinetics. When ABTS is oxidized by laccase, it changes from nearly colorless to deep greenish‐blue with an absorbance peak at 405 nm. ABTS may be used at different concentrations (0.02–9.1 mM) in phosphate buffer (25–100 mM) and pH 5–7 24, 25. In this work, a syringe pump was connected to the flow‐through device to feed it with ABTS solution at varying flow rates. With higher flow rates, the solution had a shorter residence time near the membrane and vice versa for lower flow rates. The average dwell time in the microreactor was calculated by dividing the microreactor volume (76 µL; see Supporting Information) by the volumetric flow rate from the pump. It is expected that increased dwell time will result in more reactions between the ABTS and immobilized enzymes on the membrane. Consequently, slower flow rates should yield higher absorption at 405 nm. Flow rates between ∼2 and 50 mL/h yielded about 5–120 s of average dwell time. The reacted solution was directed into a cuvette and analyzed in an optical spectrophotometer for absorbance measurements. For this purpose, a USB2000 spectrophotometer running on the Oceanview spectroscopy software (both from Ocean Optics) was used with an UV LED as the light source. The overall setup is depicted in Figure 7.

Concentration of the reacted ABTS solution was calculated by rearranging the Beer‐Lambert law — as presented in Equations (1) and (2). Here, *c*_0_ is molarity of the ABTS used, *c* is the concentration of the reacted ABTS solution, *ε* is the molar absorption coefficient (36 000 M^−1^cm^−1^ for ABTS), and *L* is the width of the cuvette, giving the absorption *A*.

(1) A=εLc

(2) $$c=\frac{A}{\varepsilon L}$$

(3) Percentage  of  reacted  molecules =100cc0

## 3 RESULTS AND DISCUSSION

### 3.1 Electrochemical detection of immobilized enzyme activity

**Figure 8:**
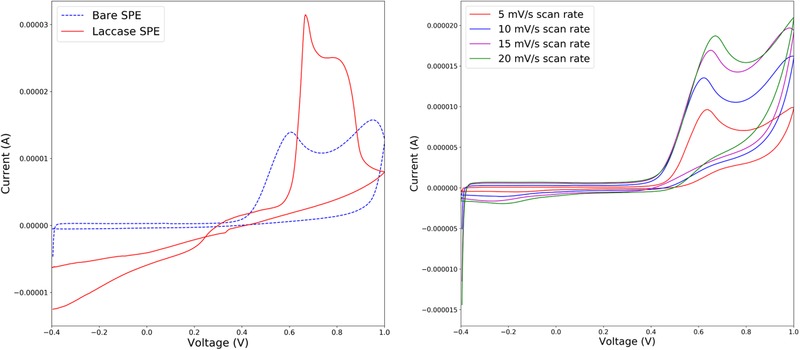
Cyclic voltammetry of $5\;\times \;10^{-4}$ mol/L m‐cresol at bare SPE and Laccase/SPE performed at 10 mV/s scan rate (left); bare SPE voltammetry performed at different scan rates in 0.01 mol/L non‐deaerated acetate buffer (right)

The electrochemical properties of cresol at bare SPEs and modified SPEs with *T. versicolor* laccase enzyme (Laccase/SPE) were investigated using cyclic voltammetry over a range of scan rates. The m‐cresol isomer provides two oxidation peaks at bare SPE in the acetate buffer solution at pH 5.0. The highest oxidation peaks observed were 13.91 and 15.75 µA, while the peak potentials of the oxidation peaks were recorded at 0.606 and 0.95 V, respectively. On the other hand, highest oxidation peak height detected for the laccase‐modified SPEs were 31.45 and 25 µA at applied potentials of 0.67 and 0.74 V, respectively. It is evident from Figure 8 (left) that the oxidation peaks are higher when the SPEs are exposed to the active laccase enzyme, which supports the development of membrane‐based lab‐on‐chip biosensors using enzymes such as *T. versicolor* laccase. The magnitude of peak currents increased with increasing scan rates as shown in Figure 8 (right).

For slow voltage scan rates, the diffusion layer will grow further from the electrode compared to faster scan rates, which produce a relatively thinner diffusion layer 26. Because of the shorter diffusion length, reactant flux to the electrode is higher at fast scan rates compared to slow scan rates. Since current is proportional to flux towards the electrode, faster scan rates increase the current produced for the voltage applied. For quasi‐reversible or irreversible cases like this instance, the peak potentials increase with the applied scan rate. Faster scan rates will encourage greater electrochemical irreversibility 26.

Cyclic voltammograms presented in Figure 8 indicate the presence of active laccase enzyme on SPE electrodes with an oxidation peak that more than doubles in current compared to enzyme‐free electrodes. This signature of an active enzyme could be used to sort biological products for their potential productivity in synthetic membrane bioreactors.

### 3.2 Absorbance measurement of immobilized enzyme activity with flowing reactant

**Figure 9:**
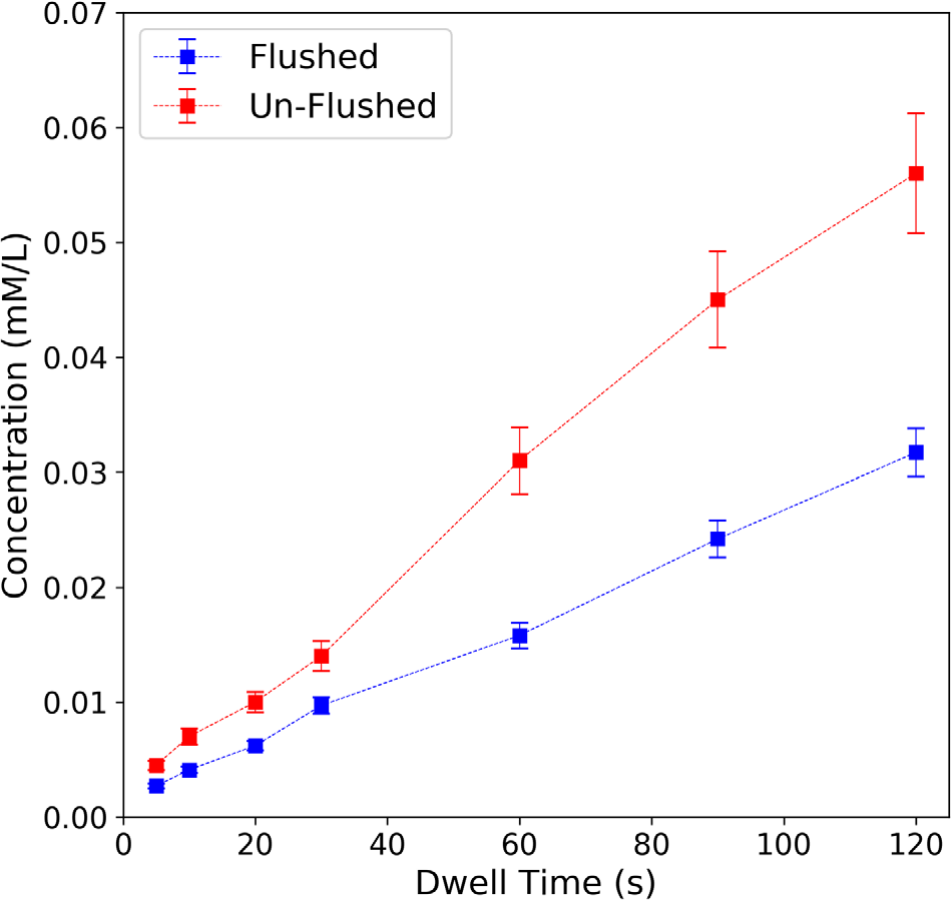
Concentration of oxidized ABTS product in the permeate solution after reacting with laccase on flushed and unflushed membranes. The same flow rate series and ABTS concentration (1 mM) was used for both reactors

Optical absorbance measurements depended on flow rate, ABTS concentration, density of immobilized enzyme, and the protocol for handling the protein‐coated membrane after drop‐casting. In order to achieve steady‐state, the flow‐through membrane reactor should be washed meticulously so that the membrane is free of loosely bound enzymes. Figure 9 shows that without flushing the reactor with DIUF water before introducing ABTS, the recorded product concentration was higher than when it was flushed. This is because the loosely bound enzymes ended up in the permeate solution and continued to oxidize ABTS. Not only did these suspended enzymes interfere with the measurement, but their departure decreased the enzyme density on the membrane, leading to a decrease in reactor efficiency over time.

**Figure 10:**
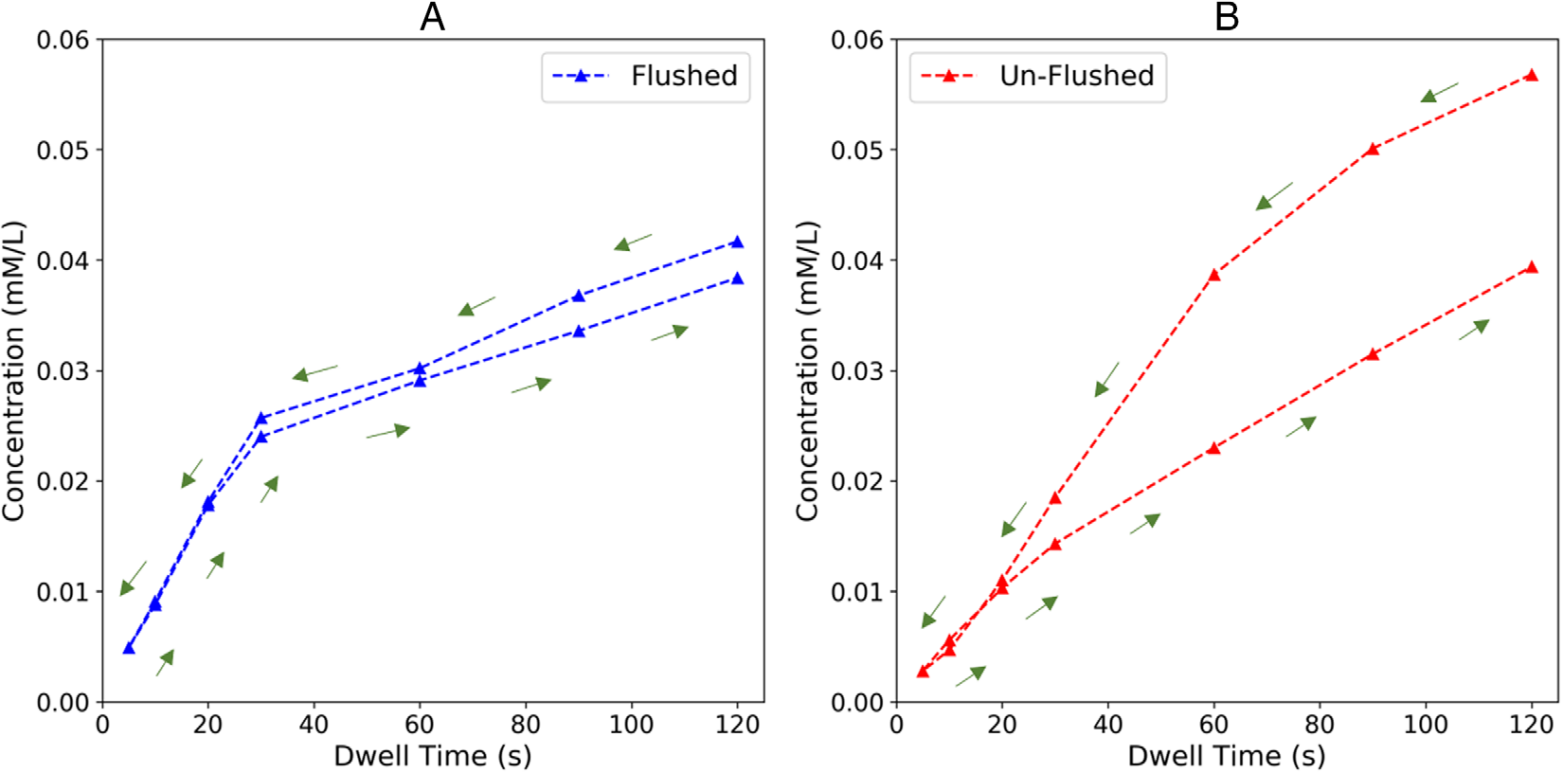
Concentration of oxidized ABTS product in the permeate solution after reacting with laccase on (A) flushed and (B) unflushed membrane reactors. The same ABTS concentration (0.5 mM) and flow rate series was used for both reactors

Figure 10 compares the effect of enzyme loss in a flushed versus an un‐flushed reactor over time, with green arrows indicating the testing sequence. At the top right of the un‐flushed plot (Figure 10, right), the product concentration is high (0.055 mM) for a dwell time of 120 s. However, after moving to higher flow rates (and shorter dwell times), then returning to the same 120 s dwell time, the reactor produces a lower product concentration (0.04 mM). Meanwhile, the round‐trip product concentration in the flushed reactor (Figure 10, left) starts out at a lower value, but is nearly reversible and is comparable to the final product concentration in the un‐flushed reactor. In either case, flushed or unflushed, longer dwell times caused reactants to spend more time in contact with enzymes, enabling more reactions to take place and increasing the concentration of oxidized product. Such round‐trip tests can not only measure enzyme activity, but help optimize the choice of flushing protocol and membrane material to promote steady‐state conditions in the reactor. These cyclic tests can also validate that old enzyme has been eluted in applications that require periodic cleaning and refreshing of the membrane reactor surface.

**Figure 11:**
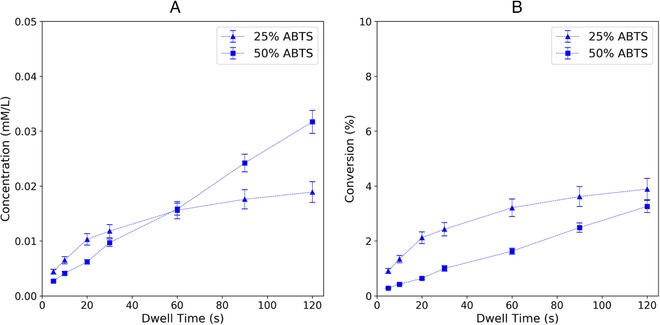
(A) Concentration of oxidized ABTS in the permeate solution after reacting with membrane‐immobilized laccase at two different ABTS concentrations (“25%” = 0.5 mM, “50%” = 1 mM). (B) Percentage of oxidized ABTS molecules over a range of dwell times at two ABTS concentrations

Besides enzyme variant, dwell time, membrane material, and flushing protocol, the reactant concentration must be optimized for maximum percent conversion in a given time. For measurement of enzyme activity, the Michaelis‐Menten model is often used to quantify the kinetic behavior of enzymes. Obtaining the Michaelis‐Menten kinetics graph requires measuring the reaction rate of the enzyme with different concentrations of substrate, in this case ABTS. This graph determines the enzyme — substrate pair's maximum catalytic efficiency (*V*_max_) and Michaelis‐Menten constant (*K*_M_). The maximum conversion rate *V*_max_ has dimensions of reactions/volume/s while *K*_M_ has dimensions of concentration and is related to binding affinity. Together, these parameters describe a particular enzyme‐substrate pair's activity independent of volume or concentration 27, and are thus useful for comparing different enzymes, but because a series of experiments must be carried out, measuring the Michaelis‐Menten values from small amounts of sample can be challenging. Towards obtaining enzyme activity parameters in a miniaturized flow‐through format with bound enzyme, Figure 11 compares two concentrations of ABTS substrate over the same flow rate series. Both 0.5 and 1 mM ABTS concentrations lead to increased product concentration with greater dwell time (Figure 11, right). The percentage conversion for the 0.5 mM reactant is higher than that for the 1 mM reactant at short dwell times, but as dwell time lengthens, the two start to converge (Figure 11, left), suggesting that access to enzyme is limiting the conversion rate. Although higher concentrations of reactant do produce more product per time, they also lead to greater amounts of wasted reactants in the effluent stream. If reactants are expensive relative to enzyme, this reactor could be redesigned to fit more membrane surface area in the same volume, putting more enzyme within a diffusion length of the reactants. Folded membranes and hollow‐fiber membrane arrays are two common area‐expanding strategies to increase the throughput of both membrane reactors and membrane filters.

## 4 CONCLUDING REMARKS

Voltammetric analysis indicates the presence of laccase on SPE electrodes with an oxidation peak that more than doubles in current compared to enzyme‐free electrodes. This signature of an active enzyme can be used to sort biological products for their potential productivity in polymeric membrane bioreactors.

To optimize not only the enzyme itself but reactant concentration, reactor geometry, membrane materials and methods, and flow rates, a colorimetric indicator (ABTS) was used to measure laccase activity in a flow‐through format. This scaled‐down system used only 60 µg of enzyme. Key developments toward a miniaturized membrane reactor optimization platform were: (1) a round‐trip dwell time test to evaluate flushing protocols and (2) conversion rate comparisons to determine whether output is limited by reactant concentration or enzyme availability. The maximum product yield of 3.9% might be increased by longer dwell times. However, the flushing results in Figure 10 also suggest that better enzyme binding will lead to a more active catalytic surface and higher conversion rates for a given dwell time in this reactor. Successful development of enzyme‐based membrane bioreactors demands that enzymes are densely attached, and also attached securely enough to withstand convective flows and washing. They must be oriented to retain their natural catalytic activity and function as much as possible. Thanks to their evolution in natural systems, enzymes work under mild temperatures and in biocompatible chemical environments. Because of these advantages, the enzyme attachment problem has been approached via adsorption, physical entrapment in polymers and sol–gels, and covalent attachment or self‐assembly onto various surfaces 28, 29, 30. Histidine‐tagged recombinant proteins are an efficient method of controlling the adhesion of a protein on surfaces functionalized with cobalt, nickel, and other metal ions 31, 32. The challenge for genetic engineers is to insert the histidine tag at a location within the protein that does not interfere with regions that are used for catalysis. This research will help investigate the activity of new genetically engineered laccase variants, both naturally‐occurring and designed with adhesion tags, in a flow‐through environment using a membrane‐based miniaturized system. The larger goal is to apply enzyme‐coated nanomembranes for efficient processing of lignin‐rich biomass into fuels and other valuable chemical products.

## CONFLICT OF INTEREST

The authors have declared no conflict of interest.

## Supporting information

Supporting Information
