# Supplementary material for: Miniaturized systems for evaluating enzyme activity in polymeric membrane bioreactors
Source: Eng Life Sci. 2019 Oct 9;19(11):749–58. doi: 10.1002/elsc.201900059 (PMC6999229; doi:10.1002/elsc.201900059)
Supplement: Supplementary file 1 — Supporting Information [file ELSC-19-749-s001.pdf]

Method of quantifying the amount of activity of the enzyme

For a better understanding of enzyme activity, its kinetics must be explained. The Michaelis-Menten model describes the kinetic behavior of many enzymes. Obtaining the Michaelis-Menten kinetics graph requires calculating the rate of reaction between our enzyme and different concentrations of ABTS. This graph will determine an enzyme – substrate (ABTS) pair's maximum catalytic efficiency (V_{max}) and Michaelis constant (K_M), which are important parameters to ascertain a particular enzyme's activity [1].

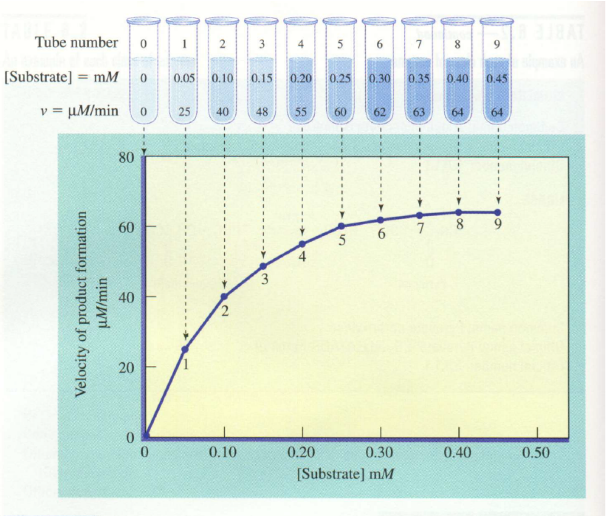

Figure 1. Michaelis–Menten saturation curve for an enzyme reaction showing the relation between the substrate concentration and reaction rate [2].

Calculating the Volume of the Microreactor

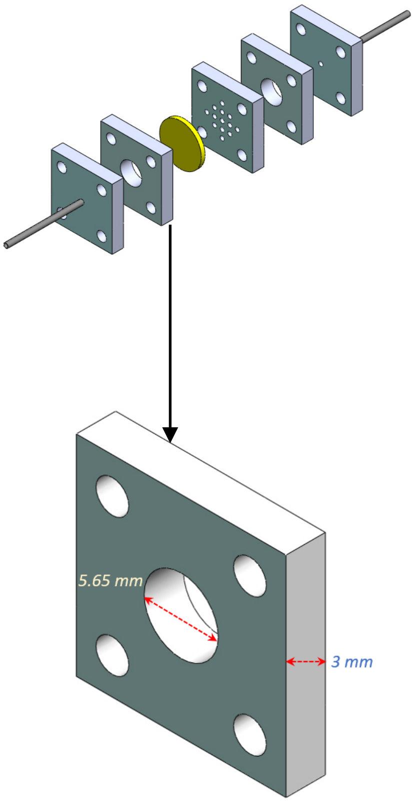

Figure 2. Cross-section of the micro-reactor reservoir.

Microreactor Volume $= \pi$ (Reservoir Radius)^2 (Reservoir Depth)

$= \pi\ (0.565/2)^2\ (0.3)$

$= 0.076\ \text{cm}^3$

$= 0.076$ ml

$= 76\ \mu$l [since, 1 ml = 1 cm^3]

References

[1] J. M. Berg, J. L. Tymoczko, L. Stryer, *Biochemistry*, W. H. Freeman and Company, New York 2002.

[2] R. F. Boyer, *Concepts in Biochemistry*, Wiley, 2006.
